# Skin Cancer and Dermoscopy Training for Primary Care Physicians: A Pilot Study

**DOI:** 10.5826/dpc.1101a145

**Published:** 2021-01-29

**Authors:** Valeria De Bedout, Natalie M. Williams, Ana M. Muñoz, Ana M. Londoño, Manuela Munera, Natalí Naranjo, Lina M. Rodriguez, Alejandra M. Toro, Feng Miao, Tulay Koru-Sengul, Natalia Jaimes

**Affiliations:** 1 Dr. Phillip Frost Department of Dermatology and Cutaneous Surgery, University of Miami Miller School of Medicine, Miami, Florida, USA; 2 Department of Dermatology, Universidad Pontificia Bolivariana, Medellin, Colombia; 3 Department of Public Health Sciences, University of Miami Miller School of Medicine, Miami, Florida, USA; 4 Sylvester Comprehensive Cancer Center, Miami, Florida, USA

**Keywords:** skin cancer, dermoscopy, dermoscopy training, diagnostic accuracy

## Abstract

**Introduction:**

The primary objective of this study was to determine the diagnostic accuracy and effect of an educational training on skin cancer course and dermoscopy use among primary care physicians in rural areas of Colombia. The secondary objective was to assess the diagnostic accuracy of skin cancer diagnosis and detection rate after 3 months of the initial training.

**Methods:**

Twenty-one primary care physicians from 6 rural areas of Colombia participated in a 2-day skin cancer and dermoscopy training, followed by a day-long hands-on session on dermoscopy at a free skin cancer screening event. Pre- and post-tests were performed using clinical and dermoscopic images to evaluate the user’s ability to diagnose and differentiate benign and malignant neoplasms. In addition, participants’ levels of confidence were assessed.

**Results:**

After the training, the sensitivity and specificity of characterizing skin lesions as benign or malignant or providing a specific diagnosis (ie, angioma, seborrheic keratosis, basal cell carcinoma, etc.) increased by 23.6% (62.9% to 86.5%; P < 0.0001) and 21% (54.7% to 75.7%; P < 0.0017), respectively. In addition, levels of confidence when diagnosing skin lesions changed from extremely low or low, to high or extremely high by 20.7% (38.3% to 59%) using dermoscopic images (odds ratio (OR) 3.22; 95% confidence interval (CI): 2.67–3.86; P < 0.0001). The secondary objective was not achieved due to loss of follow-up of the majority of participants.

**Conclusion:**

Providers serving populations with limited healthcare access may benefit from education in diagnosing and differentiating skin cancer with the use of dermoscopy, which may ultimately improve patient care and reduce healthcare costs.

## Introduction

Primary care physicians (PCPs) play a critical role in the health system of many Latin American countries. PCPs are usually the first physicians to encounter the patient and are responsible for the decision-making as to whether or not a patient needs further evaluation. Among dermatologic conditions, skin cancer continues to be a public health burden worldwide and most cases can be cured if detected early. Thus, early detection is paramount in improving patient prognosis and quality of life, while reducing healthcare costs. Various strategies, both in primary and secondary prevention, have been suggested and implemented including education for medical and non-medical communities. In fact, different educational interventions for PCPs have been developed within the field of skin cancer, but few have been carried out in Latin America [1].

Dermoscopy is a noninvasive, in-vivo imaging technique that allows the visualization of subsurface structures of the skin that are otherwise not visible to the naked eye [2,3]. Traditionally, dermoscopy has been used by, and taught to, dermatologists. However, during the last decade its use has been explored and extended to PCPs [4–6]. Although dermoscopy increases the diagnostic accuracy of skin cancer diagnosis, this improvement is contingent on acquiring dermoscopy training. Without training, the use of dermoscopy may result in poorer performance compared to clinical examination [7–9]. It has been shown that PCPs who are trained in dermoscopy not only improve their sensitivity for the diagnosis of skin cancer, but also reduce the number of unnecessary biopsies and referrals [4–6]. Oftentimes, there is a lack of specialized medicine (eg, dermatology) in underserved populations including rural areas of countries such as Colombia. In these populations the PCP may represent the first, and sometimes the only, healthcare provider. Thus, having PCPs trained in diagnosing and differentiating skin cancer using dermoscopy would be an efficient strategy to improve the early detection of skin cancer and consequently reduce morbidity and associated healthcare costs.

The present pilot study developed a skin cancer and dermoscopy training intervention for PCPs in the Eastern rural region of the Department of Antioquia in the country of Colombia. The Department of Antioquia is the second most populated Department of Colombia among its 32 departments, with an estimated population of 6.4 million. Antioquia is located in the central northwestern part of Colombia with most of its territory being part of the Andes mountain range. The racial background of this region is largely mestizo and white. These individuals are more prone to developing skin cancer because of their skin phenotype, increased ultraviolet radiation (UVR) exposure due to their frequent outdoor activities (eg, agriculture), and the geographic location of their municipalities at high altitudes with high UVR indexes year-round [10]. The primary objective of this study was to determine the diagnostic accuracy and effect of an educational training on skin cancer and dermoscopy use among PCPs in this rural region of Colombia. The secondary objective was to assess the diagnostic accuracy of skin cancer diagnosis and detection rate after 3 months of the initial training.

## Methods

### Study design and population

PCPs from hospitals of 6 municipalities in the rural area of the Eastern Region of Antioquia, Colombia (ie, Alejandría, El Peñol, Guatapé, Marinilla, San Carlos, and San Rafael) were invited to participate in the study in November of 2018. This area was selected given its geographic location combined with its low healthcare access, offering level 1 or 2 medical services without specialized medicine. The total population for these 6 municipalities is 111,175. The study was IRB-approved at the Universidad Pontificia Bolivariana in Medellín, Colombia.

### Intervention: Training in Skin Cancer and Dermoscopy

**Figure 1:**
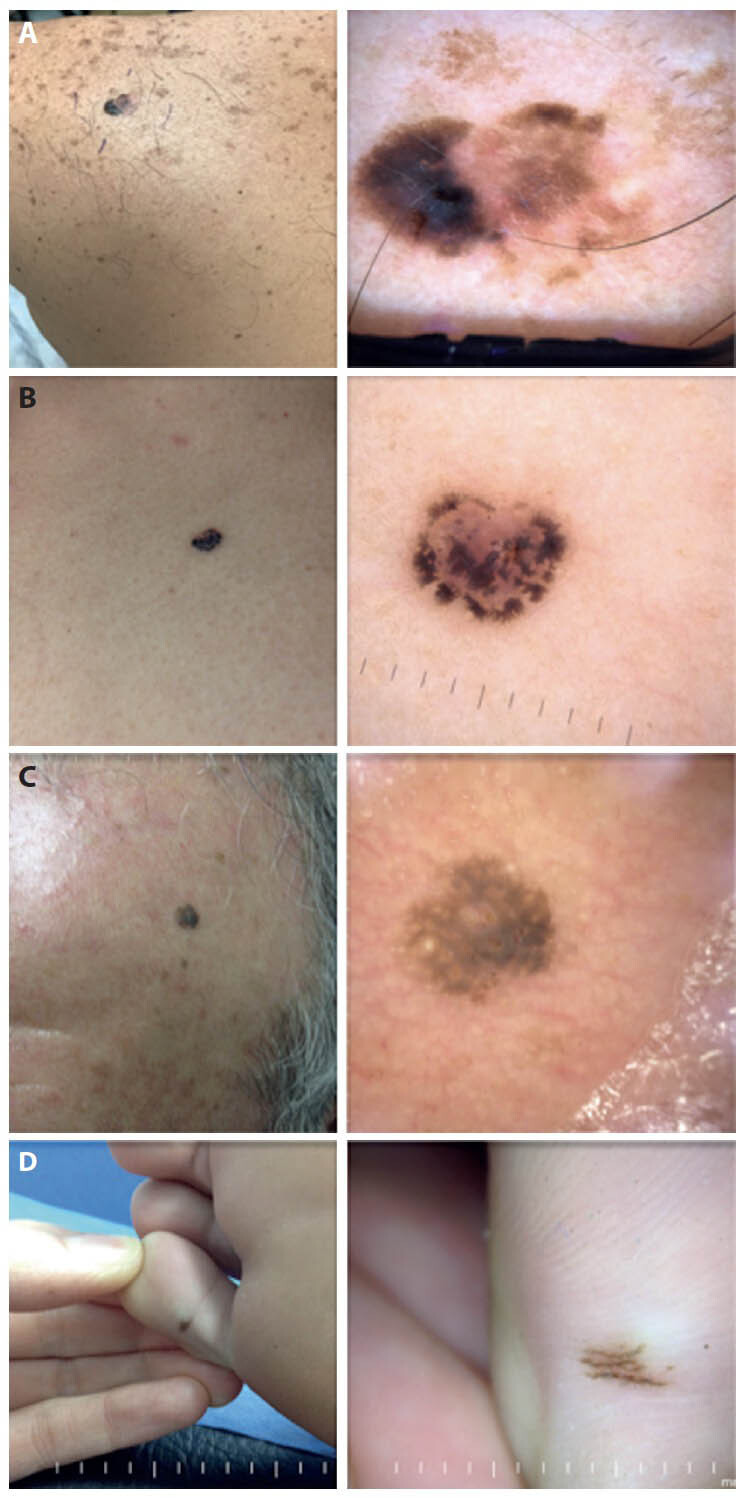
Test images. Clinical and dermoscopic images of various skin lesions were presented separately during the pre-test and post-test. For each case, a clinical image (left) was presented followed by the clinical and dermoscopy (right) image: (A) melanoma, (B) basal cell carcinoma (C) seborrheic keratosis, and (D) acral nevus.

Inclusion criteria consisted of healthcare professionals working in primary care settings who were willing to voluntarily participate in the study. Individuals who did not complete both pre- and post-tests or did not attend at least 50% of the course were excluded. PCPs participating in the study received a 2-day course on the theoretical and practical aspects of diagnosing and differentiating skin cancer clinically and with dermoscopy. Training was provided by 5 dermatology residents and 2 dermatologists with expertise in dermoscopy. A quasi-experimental study with a pre-test/post-test design was performed. A total of 50 cases were presented on a large screen using PowerPoint before and after training. First, the clinical images of all 50 cases (24 benign, 26 malignant) were presented. For each case, 3 questions were asked: 1) Is the lesion benign or malignant? 2) What is the possible diagnosis (seborrheic keratosis [SK], nevus, squamous cell carcinoma [SCC], melanoma, or other)? and 3) What is the level of confidence (on a scale from 1 to 5, with 1 being not confident and 5 being extremely confident)? Examiners recorded their responses by writing their answer choice on a paper-based test. After completion of the presentation of clinical images, the cases were presented again with the addition of a dermoscopic image as demonstrated in Figure 1, and the same 3 questions were answered. After the 2-day course, the same 50 cases were presented using the same format and questions (post-test). Each trainee was given 1 minute to respond to each case in both the pre- and post-examinations. Additionally, participants were instructed not to discuss the cases after the pre-test. A few of the test images were included by way of illustration or discussion in the teaching activities. Subsequently, participants joined a 1-day hands-on session with one-on-one training during a free skin cancer screening event offered to local communities. Each participating institution was provided with a dermoscope to use after training.

#### Statistical Analysis

Descriptive statistics were used to describe the demographic information and other characteristics of the study participants including gender, type of practice (private vs public), years of practice, average of cases of dermatologic conditions seen per months, and knowledge about dermoscopy, self-skin examination, and full body skin examination. To evaluate the training intervention, pre-test and post-test results were measured. Measures of validity included the sensitivity and specificity of diagnostic accuracy, and Kappa statistics for agreement and overall percent agreement. Dichotomous outcome measurements were created using the data provided by the participants. Variables included: 1) benign vs malignant and 2) possible diagnosis. The values of these variables were used to create cross-classifications of benign and malignant lesions and to calculate overall sensitivity, specificity, overall agreement and Kappa statistics with 95% confidence intervals (CI) for both pre-test and post-test. To evaluate the improvement after the 2-day course, we used McNemar’s test to compare sensitivities, specificities, and overall agreement between pre-test and post-test. The association between diagnosis confidence and training were estimated by odds ratio (OR) with corresponding 95% CI and P value. Data management all of the statistical analyses were carried out using SAS v9.4 (SAS Institute Inc., Cary, NC, USA).

#### Three-Month Follow-up

To evaluate the longer-term effects of the intervention, a 3-month follow-up of the PCPs was planned to learn whether the training was serving the target population. For this phase of the study, an online rehearsal course in dermoscopy was offered to the participating PCPs. In addition, PCPs and hospitals were asked to provide a de-identified list of patients with skin cancer diagnoses that were seen by the participating PCPs 3 months prior to and 3 months after the training course. For this list, the International Statistical Classification of Diseases and Related Health Problems 10^th^ Revision codes (ICD-10) were used.

## Results

A total of 21 PCPs from 6 hospitals of 6 municipalities participated in the course. Two physicians were present for less than half of the course; therefore, 19 PCPs were included in the final analysis. Demographics of the PCPs are listed in Table 1, and information on their current understanding and clinical practice with respect to skin cancer is listed in Table 2.

### Two-Day Skin Cancer and Dermoscopy Training

#### 1. Benign vs Malignant

**Figure 2:**
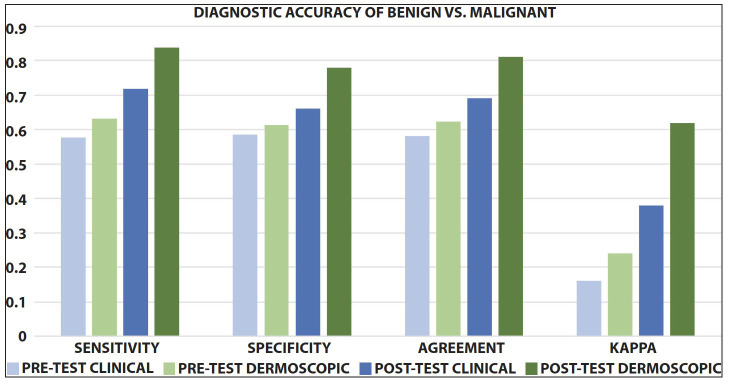
Diagnostic accuracy of benign vs malignant. An increase in sensitivity and specificity in differentiating skin lesions as benign vs malignant was observed after the two-day skin cancer and dermoscopy training.

The PCPs ability to differentiate malignant lesions (ie, melanoma, BCC, and SCC) from benign lesions (ie, nevus, dermatofibroma, angioma, and SK) was tested and analyzed using pre- and post-test evaluations. Of the test cases, 48% of lesions were benign. Pre- and post-test results are demonstrated in Table 3. The sensitivity for skin cancer diagnosis using clinical images alone was 57.8%, improving to 71.8% (P < 0.0001) using clinical images alone, and 83.9% (P < 0.0001) using clinical and dermoscopy images. The specificity also increased from 58.5% to 66.1% (clinical images alone, P = 0.0107) and 78% (clinical and dermoscopy images, P < 0.0001) (Figure 2). Overall, after the educational intervention, the PCPs’ ability to accurately identify lesions as benign or malignant significantly improved.

#### 2. Specific Diagnosis

**Figure 3:**
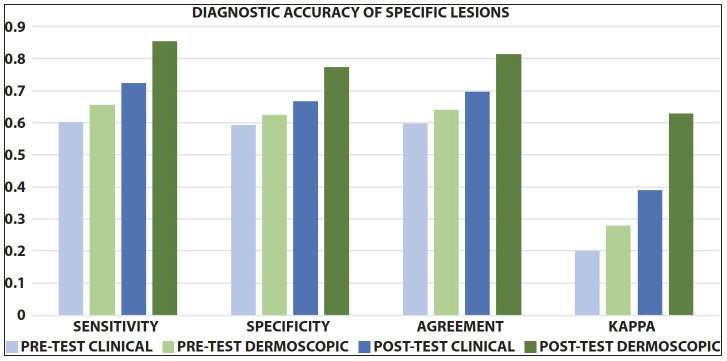
Diagnostic accuracy of specific lesions. An increase in sensitivity and specificity in diagnosing specific skin lesions was observed after the two-day skin cancer and dermoscopy training.

The PCPs ability to specifically diagnose benign (ie, angioma, nevus, dermatofibroma, SK) or malignant lesions (ie, BCC, SCC, melanoma) was tested and analyzed using pre- and post-test evaluations. Of the test cases, 22% were melanoma, 20% BCC, 10% SCC, 18% nevi, 10% SK, 8% solar lentigo, 4% dermatofibroma and hematoma, and 2% angioma and angiokeratoma. The sensitivity for any skin cancer using clinical images alone was 60.1%, improving to 72.4% (clinical images alone, P < 0.0001) and 85.4% (clinical and dermoscopy images, P < 0.0001). The specificity increased from 59.4% to 66.8% (clinical images alone, P = 0.012) and 77.3% (clinical and dermoscopy images, P < 0.0001) (Figure 3).

#### 3. Confidence

**Figure 4:**
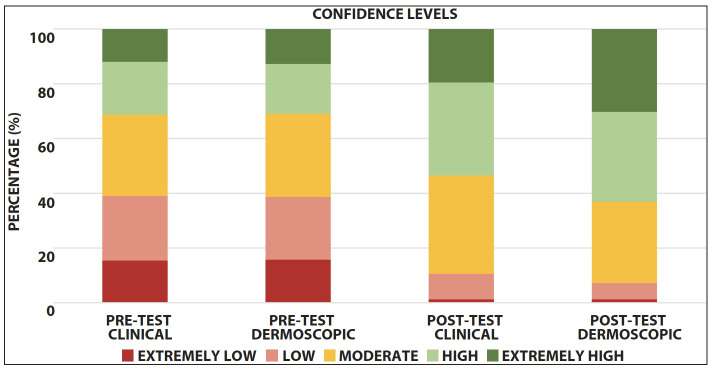
Confidence levels among participants. An increase in physician confidence in diagnosing skin lesions was observed after the two-day skin cancer and dermoscopy training.

After the educational intervention, the number of participants who labeled their level of confidence when diagnosing skin lesions as “high” or “extremely high” increased by 19% (30.9% to 49.8%; OR: 2.22; 95% CI: 1.84–2.67; P < 0.0001) for clinical images, and 28% (30.9% to 59%; OR: 3.22; 95% CI: 2.67–3.86; P < .0001) for dermoscopic images (Figure 4). Furthermore, confidence was correlated with diagnostic accuracy. Considering confidence as a continuous measurement from 1 (“extremely low”) to 5 (“extremely high”), the correct diagnosis rate increased by 34.4% for every 1-point increase in confidence (overall OR: 1.34; 95% CI: 1.27–1.43). During the skin cancer screening, the majority of participants continued to report high levels of confidence (53.2% for clinical images, 73.7% for dermoscopic).

##### Three-Month Follow-up

To evaluate the effects of the intervention on the participants’ clinical practices, a 3-month follow-up of the PCPs was planned to learn whether the training was serving the target population. However, most of the participating PCPs were lost to follow-up, and the lists with ICD-10 codes were only provided by 2 of the participating physicians.

## Discussion

Timely and accurate skin cancer diagnosis continues to be a clinical challenge, especially in underserved populations, rural areas, and developing countries where resources and geographic locations limit access to specialized healthcare. The use of dermoscopy increases the diagnostic accuracy of skin cancer by revealing structures and features otherwise invisible to the naked eye [11]. In the primary care setting, dermoscopy has been shown to improve the diagnostic accuracy for skin cancer and enhance the capacity of PCPs to appropriately triage skin lesions [3,12–15]. A randomized clinical trial found that the probability of correctly diagnosing skin lesions was 1.25 times higher in PCPs using dermoscopes compared to PCPs using only naked-eye examination [16]. Training PCPs in dermoscopy not only advances physician knowledge and diagnostic skills, but can also benefit their community, where he or she may be the only healthcare provider. In our study, the 6 hospitals served about 111,175 persons, all of whom resided in municipalities where PCPs were the only healthcare providers.

Studies have revealed that mastery-learning courses in dermoscopy led by dermatologists improve diagnostic accuracy, increase physician confidence, and decrease referrals of benign lesions [17–21]. Similarly, our study demonstrated that PCPs increased their sensitivity and specificity for detecting skin cancer after participating in the 2-day course on skin cancer and dermoscopy. This highlights the already known utility of dermoscopy in the detection of skin cancer by PCPs and emphasizes the impact that an educational training program may have, even when it is as short as a few days. On the other hand, confidence, which is defined as the degree of certainty in the correctness of a diagnosis, is usually influenced by a variety of factors, including experience, level of training, and self-assurance [22]. The addition of dermoscopy to the clinical examination has been shown to reduce doubt and increase confidence, especially when evaluating skin lesions that are clinically challenging, but clearly benign or malignant under dermoscopy [22]. Our results demonstrate that confidence improved among the majority of participating PCPs after being trained in skin cancer and dermoscopy, and was correlated with increased diagnostic accuracy. This also suggests that confidence and knowledge can be further consolidated and maintained over time when additional rehearsals or trainings occur.

Conventionally, skin cancer diagnosis and dermoscopy has been taught through traditional lectures and problem-based learning using images of skin lesions. In this study we used a combined approach incorporating passive (ie, traditional lectures) and active (ie, problem-based) learning strategies, followed by experiential learning with live patient encounters (LPE) during a skin cancer screening event. Although there are several forms of active learning, we included problem-based learning and experiential learning with LPE in a one-on-one training as an approach to meet the 4 key requirements of active learning: 1) activating prior knowledge; 2) involving the majority of students; 3) promoting metacognition to increase awareness of strengths and weaknesses as learners; and 4) providing participants with feedback about their learning [23]. LPE is an active learning strategy rated by students as better than problem-based learning, which can result in increased performance and learning [24]. This one-on-one training ensured that the providers felt comfortable handling and using a dermoscope in a real clinical setting before incorporating it in their clinical practice.

Our study has several limitations. First, we acknowledge that this was a pilot study, the number of participants was small, and the sample was a non-probability-based convenience population, selected based on the geographic location of the 6 municipalities. Thus, our sample may not represent the target population. Second, the secondary objective of the study was to follow up with the PCPs after 3 months for reevaluation and to record the number of skin cancer diagnoses made since the initial training. However, this objective proved to be challenging since the majority of participants were lost to follow-up. Possible reasons for this include a lack of interest and the temporality of some of the providers, as many work for less than a year during their social service. Therefore, we suggest that educational initiatives abroad should involve local academic or governmental entities that can implement and maintain such programs in a more rigorous form. Another alternative would be to provide the educational initiative as part of the curriculum in medical schools during the last year of training (eg, internship). Furthermore, in the demographic survey related to skin cancer practices and knowledge, a large proportion of questions went unanswered by PCPs, limiting the results of this questionnaire.

## Conclusions

PCPs play a key role in healthcare across the globe, especially those of developing countries and rural areas, including Latin America. We conclude that appropriate training in skin cancer diagnosis and dermoscopy with active learning strategies can increase physician knowledge and confidence. This may ultimately decrease healthcare costs by reducing the number of unnecessary referrals, while improving the early detection of skin cancer in underserved areas lacking healthcare specialists.

## Figures and Tables

**Table 1:** Characteristics of the Healthcare Professionals in the Study

Participant Characteristics	n	%
**All**	21	100.0
**Gender**		
Female	11	52.4
Male	10	47.6
**Job type of the healthcare professional**		
MD	18	85.7
Nurse	1	4.8
Other	2	9.5
**Where do you work?**		
Unanswered	3	14.3
Community hospital	11	52.4
Private hospital	1	4.8
Private practice	6	28.6
**Years of practice**		
Unanswered	2	9.5
<5	15	71.4
>5	4	19.0

**Table 2:** Skin Cancer Knowledge and Practices of Healthcare Professionals in the Study

Participant Skin Cancer Knowledge and Practices	n	%
**All**	21	100.0
**Number of dermatology conditions seen per week**		
Unanswered	9	42.9
1	3	14.3
2	2	9.5
3	2	9.5
4	1	4.8
5	3	14.3
20	1	4.8
**Number of dermatology conditions seen per month**		
Unanswered	11	52.4
1	1	4.8
2	1	4.8
3	2	9.5
5	2	9.5
6	1	4.8
10	1	4.8
20	2	9.5
**Cases of skin cancer seen per month**		
Unanswered	7	33.3
0	5	23.8
1	5	23.8
2	3	14.3
3	1	4.8
**Do you know what a dermatoscope is?**		
No	0	0.0
Yes	21	100.0
**Have you used a dermatoscope?**		
No	14	66.7
Yes	7	33.3
**Have you heard of self-skin exams?**		
No	8	38.1
Yes	13	61.9
**Do you discuss self-skin examinations with patients?**		
Unanswered	9	42.9
No	6	28.6
Yes	6	28.6
**How often should self-exams be performed?**		
Unanswered	15	71.4
Monthly	2	9.5
Once a year	2	9.5
Other	1	4.8
Weekly	1	4.8
**Do you know the ABCDs of melanoma?**		
No	4	19.0
Yes	17	81.0
**Do you discuss sun protection measures with patients?**		
Unanswered	2	9.5
Yes	19	90.5
**How often do you perform full body skin exam on your patients?**		
Unanswered	6	28.6
Never	3	14.3
Every visit	2	9.5
Once a year	2	9.5
Twice a year	1	4.8
Other	7	33.3

**Table 3:** Participant Responses on Pre- and Post-Tests

	Pre-Test Clinical		Pre-Test Dermoscopic		Post-Test Clinical		Post-Test Dermoscopic	
	n	%	n	%	n	%	n	%
All images	1,000	100.0	1,050	100.0	952	100.0	952	100.0
Accuracy (benign vs malignant)								
Correct	282	28.2	305	29.0	324	34.0	429	45.1
Incorrect	702	70.2	743	70.8	561	58.9	462	48.5
Unanswered	16	1.6	2	0.2	67	7.0	61	6.4
Nature of lesion								
Benign	487	48.7	519	49.4	409	43.0	409	43.0
Malignant	502	50.2	528	50.3	478	50.2	482	50.6
Unanswered	11	1.1	3	0.3	65	6.8	61	6.4
Diagnosis								
Melanoma	282	28.2	285	27.1	288	30.3	241	25.3
BCC	115	11.5	137	13.0	107	11.2	137	14.4
SCC	98	9.8	106	10.1	81	8.5	111	11.7
Nevus	207	20.7	228	21.7	174	18.3	184	19.3
SK	79	7.9	65	6.2	71	7.5	69	7.2
Dermatofibroma	35	3.5	60	5.7	39	4.1	52	5.5
Angioma	95	9.5	98	9.3	65	6.8	41	4.3
Other benign	73	7.3	69	6.6	60	6.3	56	5.9
Unanswered	16	1.6	2	0.2	67	7.0	61	6.4

* In this table, each of the 50 images were reviewed by up to 21 participants (maximum of 1,050 images)

BCC = basal cell carcinoma; SCC = squamous cell carcinoma; SK = seborrheic keratosis.
